# A case report on non‐metastatic Ewing sarcoma of the lumbar spine in a young patient

**DOI:** 10.1002/cnr2.1725

**Published:** 2022-10-03

**Authors:** Shobha Mandal, Srijana Baniya, Dipesh Kumar Rohita, Gopal Kumar Yadav, Philip Lowry

**Affiliations:** ^1^ Department of Internal Medicine Guthrie Robert Packer Hospital Sayre Pennsylvania USA; ^2^ Department of Internal Medicine Penn State College of Medicine Hershey Pennsylvania USA; ^3^ Department of Internal Medicine BP Koirala Institute of Health Sciences Dharan Nepal

**Keywords:** case report, Ewing sarcoma, laminectomy, lumbar spine, spinal neoplasm

## Abstract

**Background:**

Ewing sarcoma (ES), the second most common malignant bone tumor after osteosarcoma in the second decade, occurs in 0.9% of cases as the primary non‐sacral form.

**Case:**

A 20‐years‐old male presented with acute paraparesis of bilateral lower limb and numbness following initial back pain for the last 6 months. Magnetic resonance imaging (MRI) of the lumbar spine revealed a 4 cm enhancing soft tissue mass at the L4/L5 vertebra extending into the spinal canal with compression of the thecal sac. The computed tomography (CT) of the chest, abdomen, and pelvis revealed aggressive lytic lesions in the L4 spinous process with soft tissue extension into the spinal canal with no other site of distant metastasis. He was treated with IV steroids (Injection dexamethasone 10 mg IV followed by 4 mg tablet dexamethasone q6h; subsequently tapered off). A core needle biopsy showed a small, round blue cell neoplasm, (suggestive of a primitive neuroectodermal) stained positive for CD99 and vimentin stain. The diagnosis of ES lumbar spine was made which was treated with surgical resection with an appropriate margin measuring 8 × 4.5 × 2.5 cm with decompression and L4/5 laminectomies, which had a negative margin in the surgical pathology report. Concomitant local radiotherapy and chemotherapy [cycles of vincristine 2 mg/m^2^, adriamycin/doxorubicin 75 mg/m^2^, cyclophosphamide 1200 mg/m^2^ (VDC) with mesna rescue alternating with cycles of ifosfamide 1800 mg/m^2^ and etoposide 100 mg/m^2^ (IE)] was started. The motor strength was regained gradually with preserved spine biomechanics and oncological control with no recurrence in 2‐year follow‐ups.

**Conclusions:**

The presentation of lumbar ES can vary from local pain and swelling to acute paraparesis. Timely diagnosis and treatment with multimodal therapy, namely, steroids for acute spinal cord compression and surgery with chemoradiotherapy for ES can improve spinal biomechanics and oncological control.

## 1 INTRODUCTION

Ewing sarcoma (ES) is the second most common primary malignant bone tumor after osteosarcoma in the second decade of life.^1^ It is more common in Caucasians and occurs less frequently in African and Asian people.^2^ Pathologically, it is a highly aggressive type of small round blue cell tumor with a poor prognosis.^3^ Ewing's sarcoma family tumor cells have the translocation t(11;22)(q24; q12) in 90% of cases and the translocation t(21;12)(22;12) in the remaining 10% of cases.^4^, ^5^ The common sites of osseous involvements are the axial skeleton (54% cases) followed by the appendicular skeleton (42% cases) and other bones.^6^, ^7^ Meanwhile, the extraosseous ES that usually arises within the axial skeleton, are seen in smaller proportions with propensity for older persons and females than the osseous ES.^8^, ^9^, ^10^

Radiographic evaluation of ES shows permeative and infiltrative destruction of the involved bone, accompanied by periosteal reaction, such as onion skin appearance and codman triangle, or calcified spicule.^11^ In the primary vertebral ES, the division of the spine into non‐sacral (cervical, dorsal, and lumbar) and sacral (sacral and coccygeal) is important because sacral ES is considered aggressive and less responsive to therapy.^2^, ^12^ On the same note, the primary non‐sacral ES occurs in 0.9% of all cases.^13^

The most common symptom of ES is low back pain followed by local swelling. Rapidly progressing paraplegia is uncommon especially in the young patient, which requires a high index of suspicion for diagnosis.^14^ To our knowledge, few sporadically reported cases of ES of the spine differ in their management and success. In this study, we describe our successful experience with a young male diagnosed with rare lumbar ES, managed with local surgical resection of the tumor with laminectomy, followed by chemotherapy and radiotherapy.

## 2 CASE PRESENTATION

A 20‐years‐old male, nonsmoker, without significant past medical history, presented to the outpatient clinic of Guthrie Robert Packer Hospital, Pennsylvania, the USA with complaints of ongoing lower back pain for 6 months. The pain was controlled with over‐the‐counter medicines (oral tablet acetaminophen 500 mg/dose, and/or oral tablet naproxen 500 mg/dose) initially but lately, in the last 2 weeks, the pain has been getting progressively worse, unresponsive to the over‐the‐counter analgesics (non‐steroidal anti‐inflammatory drugs) and was associated with rapid onset bilateral lower extremities weakness and numbness. He also complained of inability to pass urine and perianal anesthesia. On physical examination, his motor strength was decreased in both lower extremities (power 3/5 each lower limb) and sensory function and reflexes were normal. The rest of the musculoskeletal and neurological examinations were within normal limits without tenderness of the hips or back. He had a low‐grade fever (37.3°C).

**FIGURE 1:**
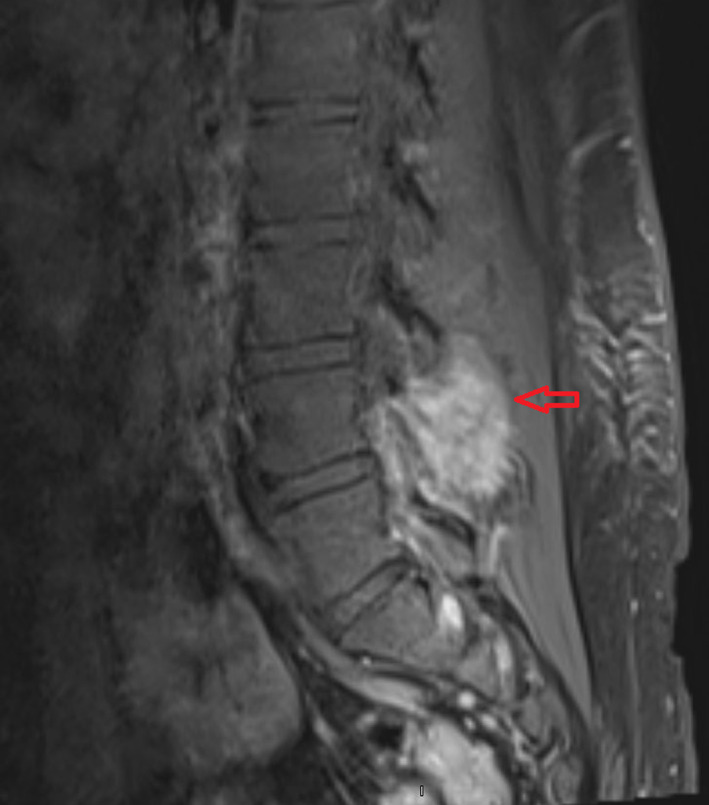
MRI of the lumbar spine showing enhancing soft tissue mass at the L4/5 vertebra extending into the spinal canal with compression of the thecal sac (red arrow).

His laboratory parameters were normal except for an elevated erythrocyte sedimentation rate (38 mm/h). X‐ray of the whole spine, chest and abdomen were normal. Magnetic resonance imaging (MRI) of the lumbar spine demonstrated a 4 cm enhancing soft tissue mass at the L4/L5 vertebra extending into the spinal canal with compression of the thecal sac without destruction of the vertebral body (Figure 1).

**FIGURE 2:**
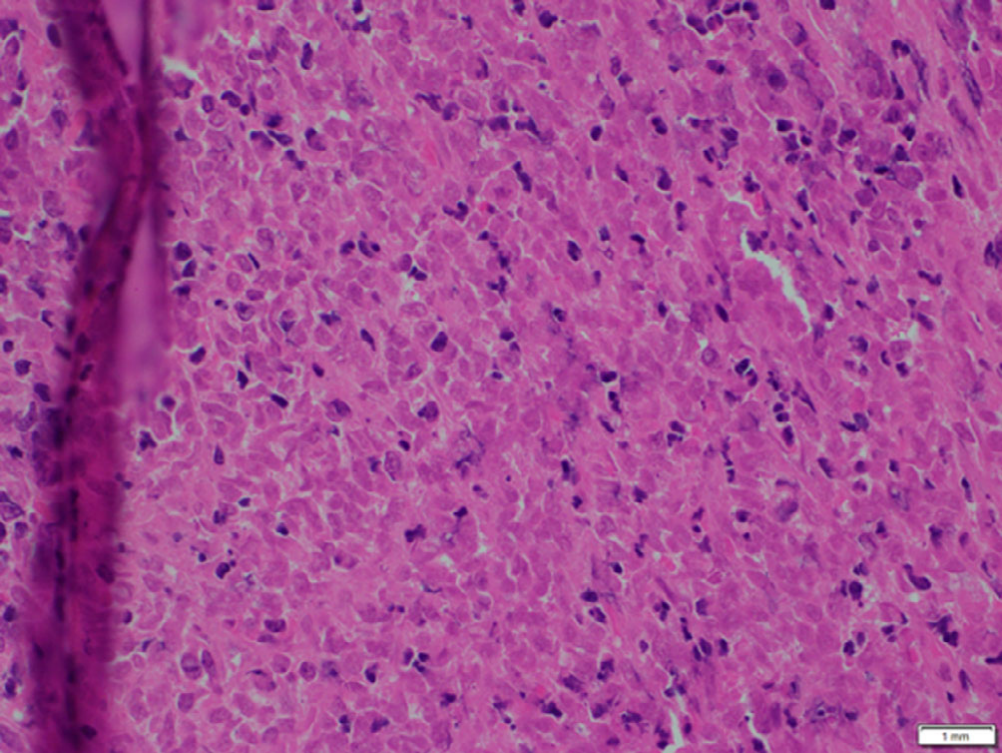
Tissue biopsy histology showing poorly differentiated small, round blue cell neoplasm suggestive of ES (40X)

He was immediately admitted and treated with intravenous steroids (Injection dexamethasone 10 mg IV followed by 4 mg tablet dexamethasone q6h; subsequently tapered off post‐operatively). Further workup with CT of the chest, abdomen, and pelvis revealed aggressive lytic lesions in the L4 spinous process with soft tissue extension into the spinal canal with no other site of distant metastasis. A core needle biopsy showed a small, round blue cell neoplasm, suggestive of a primitive neuroectodermal tumor (Figure 2).

**FIGURE 3:**
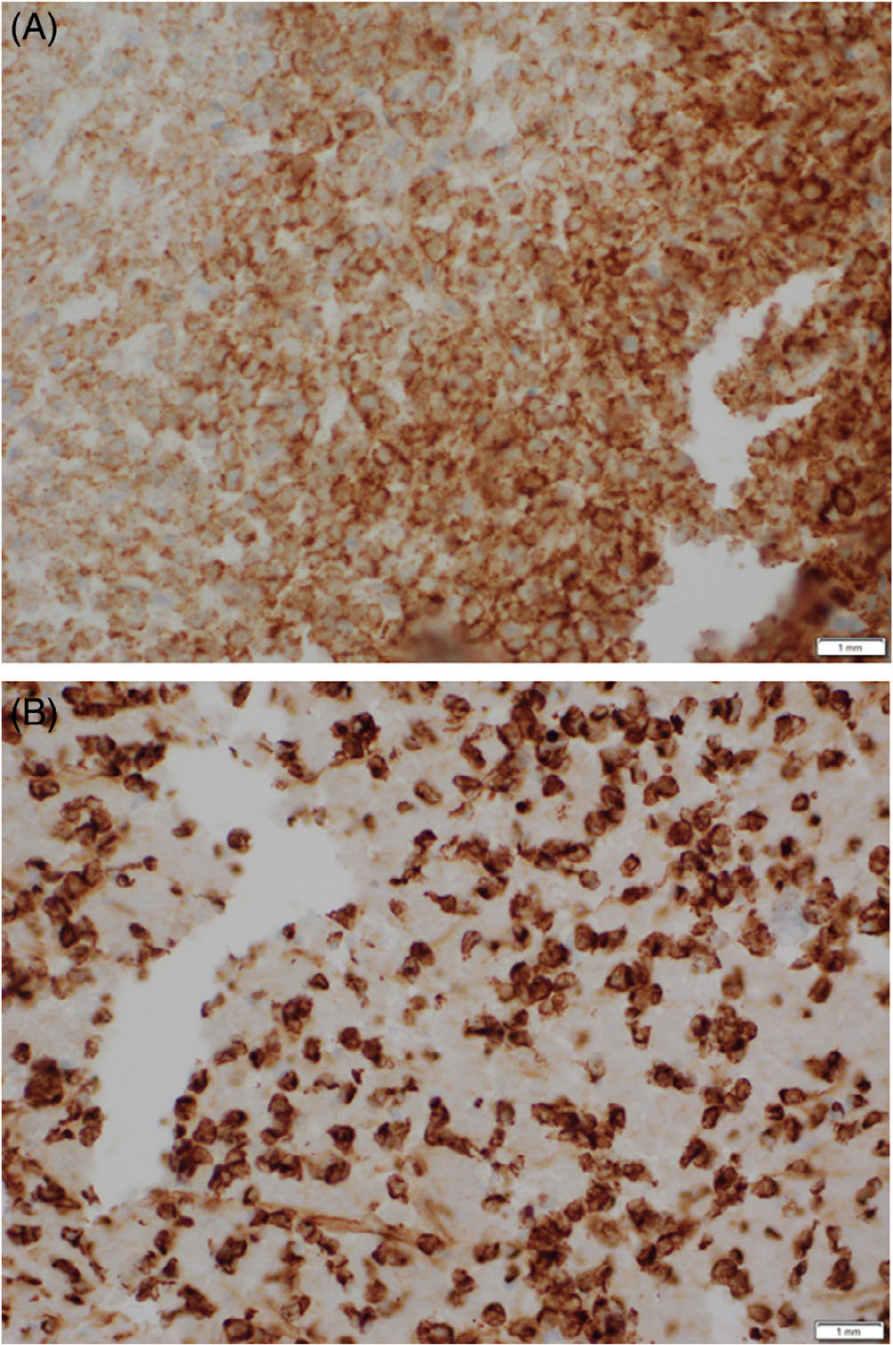
A. Immunohistochemistry of core needle biopsy; positive for CD99 stain (40X). B. Immunohistochemistry of core needle biopsy; positive for vimentin (40X)

The tumor was markedly positive for CD99 stain (MIC2) (Figure 3A) and vimentin stain (Figure 3B).

**FIGURE 4:**
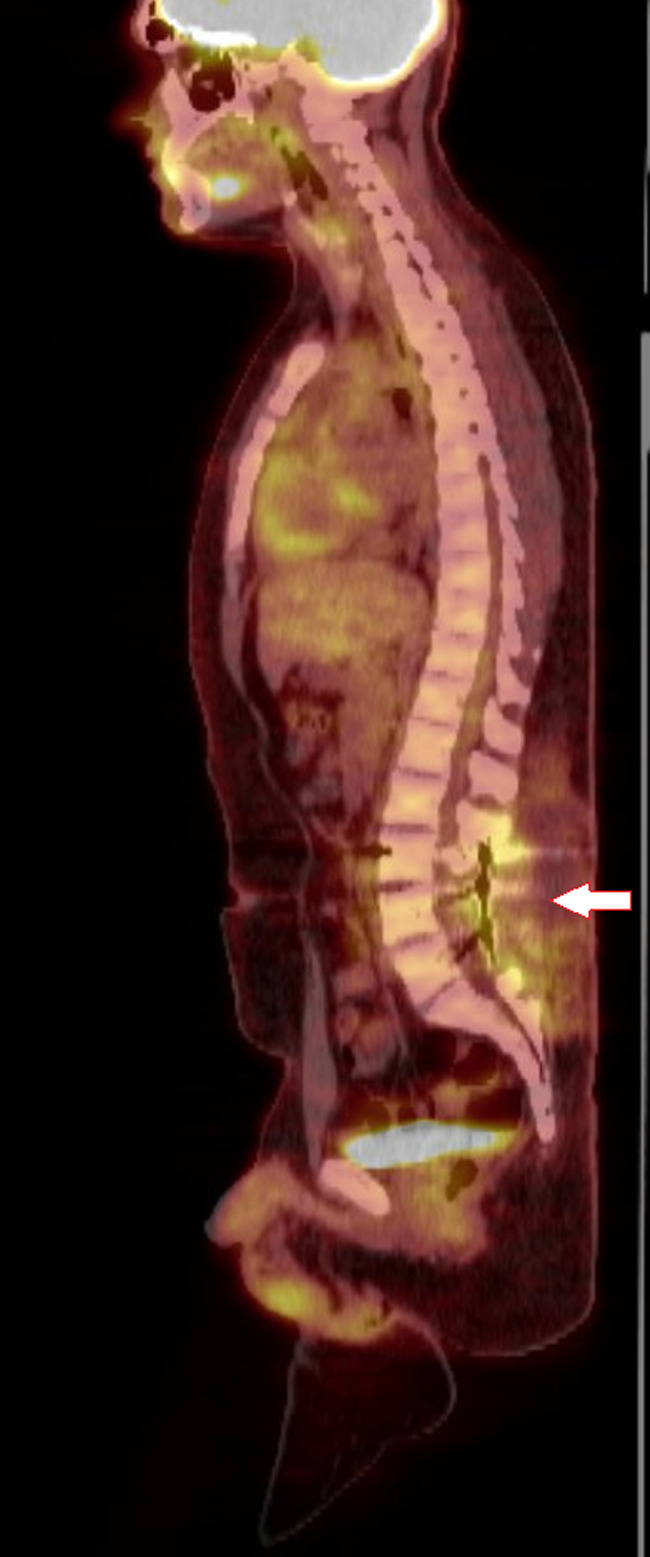
The PET CT scan showed post‐procedure changes with dorsal seroma without focal uptake

The patient complained of worsening weakness of the lower extremities; hence, he underwent primary resection of tumor measuring 8 × 4.5 × 2.5 cm with decompression and L4/5 laminectomies. Post resection, he continued to have right lower extremity numbness but reported improvement in daily activity performance. The final surgical pathology of the resected spinal mass showed a malignant small round cell tumor, 6 mitoses/10 hpf, grade 3, poorly differentiated, pT1 pNx, with negative margins. Additional molecular studies were done and he came positive for the EWSR1 gene on chromosome 22 with t(11;22) (24q;12q), consistent with the diagnosis of ES. His postoperative positron emission tomography (PET) CT scan showed post‐procedure changes with dorsal seroma without any focal uptake to suggest a residual tumor (Figure 4).

After 3 weeks from surgery, our patient was subsequently treated with 14 cycles of vincristine 2 mg/m^2^, adriamycin/doxorubicin 75 mg/m^2^, cyclophosphamide 1200 mg/m^2^ (VDC) with mesna rescue alternating with ifosfamide 1800 mg/m^2^ and etoposide 100 mg/m^2^ (IE). He tolerated the treatment well in the initial few cycles, but later, he had a few episodes of neutropenic fever requiring hospitalization. Concomitant radiotherapy was also started after cycles of chemotherapy. He received 5040 cGy radiotherapy (180 cGy per fraction, over 5.5 weeks) to tumor bed (L3/L4 spine level) with volumetric modulated arc therapy (VMAT) technique.

**FIGURE 5:**
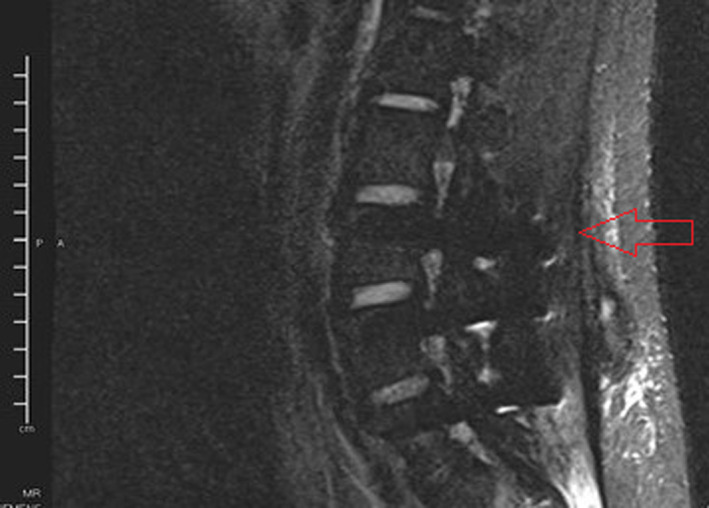
Postoperative MRI lumbar spine showing L3, L4, and L5 vertebral body fusion; L3 laminotomy; L4/L5 laminectomies; without any enhancement, foraminal narrowing, or disc herniation or alignment changes

Two‐year post‐follow‐ups, the spine biomechanics were maintained without pain flare‐ups of the lower back. There was full recovery in the strength of the lower limbs. He had no evidence of disease on a repeat MRI lumbar spine two‐year after the surgery (Figure 5).

## 3 DISCUSSION

Ewing sarcoma is a malignant, small, round cell sarcoma that arises from the primitive neuroectodermal cells that James Ewing first described in 1921.^15^ It most often affects children more than adults. Our patient who was young and had lower back pain for the preceding 6 months, presented in emergency with acute paraparesis of bilateral lower limbs, perianal anesthesia and inability to void. The features were suggestive of spinal cord decompression. The available studies showed that the only indicator for primary ES of the spine might be the spinal cord compression.^16^, ^17^, ^18^, ^19^

The mainstay of diagnosing ES, that is, small round cell tumors, is histopathology owing to its non‐presentation.^20^ Our patient underwent evaluation with immunohistochemistry (CD‐99 and vimentin) and cytogenetic analysis. CD‐99 (MIC2; single‐chain type‐1 glycoprotein) is an O‐glycosylated transmembrane protein (32 kDa) present on leukocytes and activated endothelium and encoded by the gene present on short arm of X and Y sex chromosomes. Because of higher expression in tumor cells of ES, it is a diagnostic marker of ES.^21^, ^22^ However, despite high sensitivity, it is a nonspecific marker owing to its expression in other tumors like anaplastic large‐cell lymphoma, lymphoblastic lymphoma, synovial sarcoma (round‐cell variant), etc.^23^ Similarly, vimentin is a constituent of intermediate filament, expressed in normal mesenchymal cells and provides resistance against stress.^24^ The ES stains strongly for vimentin compared to lymphoblastic lymphoma.^25^

ES is a systemic disease requiring local therapy, that is, resection with good margin and radiotherapy for local disease control and chemotherapy for systemic control.^26^, ^27^ This ES is a disease that has the micro‐metastatic disease at diagnosis and disastrous prognosis for this with macro‐metastatic disease.^27^ The chemotherapy agents vincristine, doxorubicin, cyclophosphamide, etoposide, ifosfamide, actinomycin D, and topotecan are used in various combinations, followed by local treatments (radiation and/or surgery).^28^ Our patient was treated with chemotherapy besides surgical resection and radiotherapy. The chemotherapy consisted of cycles of vincristine, adriamycin/ doxorubicin and cyclophosphamide (VAC) with mesna recue alternating with cycles of ifosfamide and etoposide (IE). This is based on the recommendations from the Children's Oncology Group that advocates the interval‐compressed therapy of VDC cycle alternating with IE cycle (every 2 weekly) derived from a randomized phase III trial. In contrast, the vincristine, doxorubicin, cyclophosphamide and actinomycin‐D (VDCA) cycle alternating with IE is also used as a chemotherapy regimen.^29^ In the study by Grier et al., the addition of IE to VDCA had significantly better five‐year relapse‐free survival compared to VDCA alone (69% vs. 54%, respectively).^30^

Our patient‐maintained spine biomechanics with no relapse or disease activity two‐year post‐surgery. An estimated 25% of patients present with metastasis at the time of diagnosis. So, the time to disease recurrence remains the important predictor of overall survival. The late recurrence (>2 years from diagnosis) is associated with longer survival than early recurrence.^31^ Similarly, elevated LDH at diagnosis, and those who underwent en bloc spondylectomy than decompression or lesionectomy had lower recurrence rates.^31^, ^32^ Therefore, multiple factors should be considered for better oncological control and preservation of spine biomechanics. Further prospective studies should be sought to generate evidence‐based treatment guidelines and the long‐term treatment effects of those modalities.

## 4 CONCLUSION

Ewing sarcoma primarily originating from the non‐sacral spine is rare in young adults. Given the rare presentation, we do not have a specific guideline on management; hence multiple modalities, including appropriate resection with a negative margin and concomitant chemoradiotherapy improved the outcome with better spine biomechanics. A high index of suspicion is required to diagnose ES non‐sacral spine in a young patient presenting with acute paraparesis following initial back pain.

## AUTHOR CONTRIBUTIONS

**Shobha Mandal:** Conceptualization (equal); data curation (equal); formal analysis (equal); funding acquisition (equal); investigation (equal); methodology (equal); project administration (equal); resources (equal); software (equal); supervision (equal); validation (equal); visualization (equal); writing – original draft (equal). **Srijana Baniya:** Funding acquisition (equal); methodology (equal); project administration (equal); supervision (equal); validation (equal); writing – original draft (equal). **Dipesh Kumar Rohita:** Data curation (equal); formal analysis (equal); funding acquisition (equal); resources (equal); software (equal); validation (equal); visualization (equal); writing – original draft (equal); writing – review and editing (equal). **Gopal Kumar Kumar Yadav:** Formal analysis (equal); resources (equal); validation (equal); writing – review and editing (equal). **Philip Lowry:** Funding acquisition (equal); investigation (equal); methodology (equal); supervision (equal); writing – original draft (equal).

## CONFLICT OF INTEREST

The authors declare no conflicts of interest.

## Data Availability

Data sharing is not applicable to this article as no new data were created or analyzed in this study.

## References

[1] Durer S , Shaikh H . Ewing sarcoma. In: StatPearls. StatPearls Publishing; 2022. Accessed May 7, 2022. http://www.ncbi.nlm.nih.gov/books/NBK559183/ 32644609

[2] Iacoangeli M , Dobran M , Di Rienzo A , et al. Nonmetastatic Ewing's sarcoma of the lumbar spine in an adult patient. Case Rep Oncol Med. 2012;2012:1, 165289‐5. 10.1155/2012/165289 PMC3485762 23133768

[3] Rajwanshi A , Srinivas R , Upasana G . Malignant small round cell tumors. J Cytol Indian Acad Cytol. 2009;26(1):1‐10. 10.4103/0970-9371.54861 PMC3167982 21938141

[4] Grier HE . The Ewing family of tumors. Ewing's sarcoma and primitive neuroectodermal tumors. Pediatr Clin North Am. 1997;44(4):991‐1004. 9286296 10.1016/s0031-3955(05)70541-1

[5] McManus AP , Gusterson BA , Pinkerton CR , Shipley JM . Review article. The molecular pathology of small round‐cell tumours—relevance to diagnosis, prognosis, and classification. J Pathol. 1996;178(2):116‐121. 8683375 10.1002/(SICI)1096-9896(199602)178:2<116::AID-PATH494>3.0.CO;2-H

[6] Stiller CA , Bielack SS , Jundt G , Steliarova‐Foucher E . Bone tumours in European children and adolescents, 1978–1997. Report from the automated childhood cancer information system project. Eur J Cancer. 2006;42(13):2124‐2135. 16919776 10.1016/j.ejca.2006.05.015

[7] Bleyer A , O' Leary M , Barr R , Ries L . Cancer epidemiology in older adolescents and young adults 15–29 years of age, including SEER incidence and survival:1975–2000. 2006. Accessed July 18, 2022. https://seer.cancer.gov/archive/publications/aya/index.html

[8] Raney RB , Asmar L , Newton WA , et al. Ewing's sarcoma of soft tissues in childhood: a report from the intergroup rhabdomyosarcoma study, 1972 to 1991. J Clin Oncol off J Am Soc Clin Oncol. 1997;15(2):574‐582. 10.1200/JCO.1997.15.2.574 9053479

[9] Pradhan A , Grimer RJ , Spooner D , et al. Oncological outcomes of patients with Ewing's sarcoma: is there a difference between skeletal and extra‐skeletal Ewing's sarcoma? J Bone Joint Surg Br. 2011;93(4):531‐536. 21464495 10.1302/0301-620X.93B4.25510

[10] Galyfos G , Karantzikos GA , Kavouras N , Sianou A , Palogos K , Filis K . Extraosseous Ewing sarcoma: diagnosis, prognosis and optimal management. Indian J Surg. 2016;78(1):49‐53. 27186040 10.1007/s12262-015-1399-0 PMC4848231

[11] Li X , Li W , Mo W , Yang Z . Acute lymphoblastic leukemia arising after treatment of Ewing sarcoma was misdiagnosed as bone marrow metastasis of Ewing sarcoma. Medicine (Baltimore). 2018;97(3):e9644. 29505001 10.1097/MD.0000000000009644 PMC5779770

[12] Ilaslan H , Sundaram M , Unni KK , Dekutoski MB . Primary Ewing's sarcoma of the vertebral column. Skeletal Radiol. 2004;33(9):506‐513. 15232658 10.1007/s00256-004-0810-x

[13] Whitehouse GH , Griffiths GJ . Roentgenologic aspects of spinal involvement by primary and metastatic Ewing's tumor. J Can Assoc Radiol. 1976;27(4):290‐297. 993244

[14] Gopalakrishnan CV , Shrivastava A , Easwer HV , Nair S . Primary Ewing's sarcoma of the spine presenting as acute paraplegia. Journal of Pediatric Neurosciences. 2012;7(1):64‐66. 22837785 10.4103/1817-1745.97630 PMC3401661

[15] Yeshvanth SK , Ninan K , Bhandary SK , et al. Rare case of extraskeletal Ewings sarcoma of the sinonasal tract. Accessed May 7, 2022. https://www.cancerjournal.net/article.asp?issn=0973-1482;year=2012;volume=8;issue=1;spage=142;epage=144;aulast=Yeshvanth 10.4103/0973-1482.95197 22531536

[16] Paul FA . Ewings sarcoma as an etiology for persistent back pain in a 17‐year‐old girl after trauma to the back. Journal of Osteopathic Medicine. 1995;95(1):58. 7860370

[17] Kogawa M , Asazuma T , Iso K , et al. Primary cervical spinal epidural extra‐osseous Ewing's sarcoma. Acta Neurochir. 2004;146(9):1051‐1053. 15340820 10.1007/s00701-004-0294-4

[18] Mukhopadhyay P , Gairola M , Sharma M , Thulkar S , Julka P , Rath G . Primary spinal epidural extraosseous Ewing's sarcoma: report of five cases and literature review. Australas Radiol. 2001;45(3):372‐379. 11531770 10.1046/j.1440-1673.2001.00942.x

[19] Electricwala AJ , Electricwala JT . Primary Ewing's sarcoma of the spine in a two‐year‐old boy. Case Rep Orthop. 2016;8(2016):e8027137. 10.1155/2016/8027137 PMC5118528 27895949

[20] Shoubash L , Nowak S , Vogelgesang S , Schroeder HWS , Müller JU . Surgical management of an adult manifestation of Ewing sarcoma of the spine—a case report. AME Case Rep. 2018;29(2):34. 10.21037/acr.2018.06.07 PMC6155648 30264030

[21] Ambros IM , Ambros PF , Strehl S , Kovar H , Gadner H , Salzer‐Kuntschik M . MIC2 is a specific marker for ewing's sarcoma and peripheral primitive neuroectodermal tumors. Evidence for a common histogenesis of ewing's sarcoma and peripheral primitive neuroectodermal tumors from MIC2 expression and specific chromosome aberration. Cancer. 1991;67(7):1886‐1893. 1848471 10.1002/1097-0142(19910401)67:7<1886::aid-cncr2820670712>3.0.co;2-u

[22] Huijbers EJM , van der Werf IM , Faber LD , et al. Targeting tumor vascular CD99 inhibits tumor growth. Front Immunol. 2019. doi:10.3389/fimmu.2019.00651 PMC6455290 31001265

[23] Chinchilla‐Tábora LM , Ortiz Rodríguez‐Parets J , González Morais I , Sayagués JM , Ludeña de la Cruz MD . Immunohistochemical analysis of CD99 and PAX8 in a series of 15 molecularly confirmed cases of Ewing sarcoma. Sarcoma. 2020;29(2020):1, e3180798‐6. 10.1155/2020/3180798 PMC7341420 32675940

[24] Satelli A , Li S . Vimentin in cancer and its potential as a molecular target for cancer therapy. Cell Mol Life Sci CMLS. 2011;68(18):3033‐3046. 21637948 10.1007/s00018-011-0735-1 PMC3162105

[25] Lucas DR , Bentley G , Dan ME , Tabaczka P , Poulik JM , Mott MP . Ewing sarcoma vs lymphoblastic lymphoma. A comparative immunohistochemical study. Am J Clin Pathol. 2001;115(1):11‐17. 11190795 10.1309/K1XJ-6CXR-BQQU-V255

[26] Thacker MM , Temple HT , Scully SP . Current treatment for Ewing's sarcoma. Expert Rev Anticancer Ther. 2005;5(2):319‐331. 15877528 10.1586/14737140.5.2.319

[27] Zöllner SK , Amatruda JF , Bauer S , et al. Ewing sarcoma—diagnosis, treatment, clinical challenges and future perspectives. J Clin Med. 2021;10(8):1685. 33919988 10.3390/jcm10081685 PMC8071040

[28] Ozaki T . Diagnosis and treatment of Ewing sarcoma of the bone: a review article. J Orthop Sci. 2015;20(2):250‐263. 25691401 10.1007/s00776-014-0687-z PMC4366541

[29] Womer RB , West DC , Krailo MD , et al. Randomized controlled trial of interval‐compressed chemotherapy for the treatment of localized Ewing sarcoma: a report from the Children's oncology group. J Clin Oncol off J Am Soc Clin Oncol. 2012;30(33):4148‐4154. 10.1200/JCO.2011.41.5703 PMC3494838 23091096

[30] Grier HE , Krailo MD , Tarbell NJ , et al. Addition of Ifosfamide and etoposide to standard chemotherapy for Ewing's sarcoma and primitive Neuroectodermal tumor of bone. N Engl J Med. 2003;348(8):694‐701. 12594313 10.1056/NEJMoa020890

[31] Leavey PJ , Mascarenhas L , Marina N , et al. Prognostic factors for patients with Ewing sarcoma (EWS) at first recurrence following multi‐modality therapy: a report from the Children's oncology group. Pediatr Blood Cancer. 2008;51(3):334‐338. 18506764 10.1002/pbc.21618 PMC2728357

[32] Samartzis D , Marco RAW , Benjamin R , Vaporciyan A , Rhines LD . Multilevel En bloc Spondylectomy and Chest Wall excision via a simultaneous anterior and posterior approach for Ewing sarcoma. Spine. 2005;30(7):831‐837. 15803089 10.1097/01.brs.0000158226.49729.6c

